# Machine learning models for the prediction of turbulent combustion speed for hydrogen-natural gas spark ignition engines

**DOI:** 10.1016/j.heliyon.2024.e30497

**Published:** 2024-05-03

**Authors:** Nelson Junior Issondj Banta, Njionou Patrick, Florence Offole, Ruben Mouangue

**Affiliations:** a National Higher Polytechnic School of Douala, University of Douala, P.O. BOX 2107, Douala, Cameroon; b Laboratory of Energy, Materials, Modeling and Method of the University of Douala, Douala, Cameroon

**Keywords:** Turbulent combustion speed, Machine learning, Hydrogen-natural gas mixture, Spark ignition engine

## Abstract

The work carried out in this paper focused on “Machine learning models for the prediction of turbulent combustion speed for hydrogen-natural gas spark ignition engines”. The aim of this work is to develop and verify the ability of machine learning models to solve the problem of estimating the turbulent flame speed for a spark-ignition internal combustion engine operating with a hydrogen-natural gas mixture, then evaluate the relevance of these models in relation to the usual approaches. The novelty of this work is the possibility of a direct calculation of turbulent combustion speed with a good precision, using only machine learning model. The obtained models are also compared to each other by considering in turn as a comparison criterion: the precision of the result, calculation time, and the ability to assimilate original data (which has not undergone preprocessing). An important particularity of this work is that the input variables of the machine learning models were chosen among the variables directly measurable experimentally, based on the opinion of experts in combustion in internal combustion engines and not on the usual approaches to dimensionality reduction on a dataset. The data used for this work was taken from a MINSEL 380, a 380-cc single-cylinder engine. The results show that all the machine learning models obtained are significantly faster than the usual approach and Random Forest (R^2^: R-squared = 0.9939 and RMSE: Root Mean Square Error = 0.4274) gives the best results. With a forecasting accuracy of over 90 %, both approaches can make reasonable predictions for most industrial applications such as designing engine monitoring and control systems, firefighting systems, simulation, and prototyping tools.

## 1 Introduction

Combustion is one of the main methods of energy production in systems present in various industrial sectors (automotive, aeronautical, aerospace, naval industry, thermal power plants, armaments, etc.). The design of equipment operating with energy from combustion requires a solid understanding in several scientific fields (modeling of turbulent flows, modeling of flames, heat transfer, chemical kinetics, etc.). Among the industrial equipment whose operation is based on the phenomenon of combustion, combustion engines are probably the most used.

With the evolution of hydrogen production technologies, hydrogen combustion in a thermal engine seems a relevant solution to replace current engines considered not environmentally friendly. Thus, several recent works focus on the characterization of the combustion of pure hydrogen [1] or mixtures with other fuels [[2], [3], [4], [5]]. In recent years, companies specializing in the design of combustion engines have faced significant challenges. Thus, on the one hand, it is a question of improving engine performance and, on the other, reducing polluting emissions with increasingly stringent environmental standards. The performance and emissions of the thermal engine are deeply related to the combustion process and, particularly, to the combustion speed. Indeed, the combustion speed directly affects the in-cylinder pressure on which the Break Mean Effective Pressure (BMEP) depends, which is one of the main characteristics of engine performance. Furthermore, the completeness of combustion, directly linked to the combustion speed, affects the production of certain pollutants, such as CO, NOx, HC, etc. The combustion speed then appears to be a key parameter for thermal engine optimization. The performance and emissions of the thermal engine are deeply related to the combustion process and, particularly, to the combustion speed.

On the other hand, the rise of clean energy and electric motors makes confrontation more difficult. Thus, many researchers are witnessing a quest for new, more energetic, and cleaner fuels [6]. In this context, hydrogen and hydrogen/natural gas mixtures will play a major role in future combustion systems. In addition, with the improvement of experimentation techniques and the evolution of data storage and processing technologies, the exploration of data-driven methods and machine learning for analyzing and modeling complex phenomena has greatly increased.

Thus, in combustion modeling with machine learning methods, L. Zhou et al. [7] offer a general review of the applications and challenges of machine learning in the field of combustion sciences for various applications. This work shows that in recent years, machine learning has had an advantage in finding hidden patterns under large amounts of data, exploring and visualizing high-dimensional input spaces, deriving complex mappings from inputs and outputs, and reducing computational cost and memory occupation. In addition, this review shows that the relationship between the input parameters involving temperature, species, and pressure and the output parameters involving chemical kinetic calculation or chemical source was established using various machine learning techniques. This work has made it possible to improve numerical simulations of the combustion phenomenon. Issondj et al. [8] and Alok W. et al. [9] worked on the Simulation of Performance and emissions-related parameters in a Thermal Engine Using a Deep Learning Approach. This work demonstrates the ability of deep neural networks to effectively predict the performance and mass flow rates of the various pollutants emitted during the operation of an internal combustion engine. Huu-Tri N. et al. [10] succeeded in the development of a machine-learning algorithm integrating chemical kinetics into the combustion numerical simulation.

Support Vector Machine (SVM) is commonly used for steady-state prediction of engine performance and pollutant emissions. SVM can model complex and nonlinear input-output relationships based on a sufficiently large training data set [[11], [12], [13]]. This approach provides a black box model without directly involving a physical understanding of the system but can be trained accurately if the model features are selected appropriately [[14], [15], [16]]. A NOx prediction model was developed for an engine running on HGNC using an optimal SVM method, whose regulation parameters were obtained with the PSO optimization method [17]. The results obtained are satisfactory, although strongly dependent on the model hyperparameters. The authors also highlighted the effect of SVM model parameters such as kernel penalty factor, insensitive band loss function, and training sample size on the quality of results. The artificial neural network (ANN) has become common in modeling combustion engines [18]. They were used to develop a model of pollutant emissions and performances for a CNG-diesel engine [19], a diesel engine coupled to an exhaust gas recirculation (EGR) system [20], a single-cylinder diesel engine fueled with diethyl ether [21], and a diesel engine fueled with animal fat. Zhentao and Jinlong [22] carried out an analysis assisted by machine learning of the performance of an ammonia engine. The results showed that the Random Forest (RF) algorithm suffered from bounds underfitting, while the algorithm effectively learned the relationship between the control variables and the engine performance. The parametric studies carried out by the machine learning model obtained suggest that the combustion law of heavy-ammonia engines is consistent with that of spark ignition engines.

Combustion speed is one of the most fundamental combustion properties. It provides a measure of the global reactivity of a fuel and helps in determining the heat release rate and the validation of detailed and reduced combustion reaction mechanisms [[23], [24], [25]]. Obtaining a quantitative model for combustion is an important problem in combustion modeling. With the rapid development of databases in combustion and computational technologies, several recent studies have focused on the use of machine learning techniques to provide a more effective solution to this problem. Thus, for the prediction of laminar flame speed in methane-air mixtures with experimental and kinetic modeling results, Jach A. et al. [26] compared three machine learning techniques, including multivariate regression models, support vector machines (SVM), and artificial neural networks (ANN). The results showed that the neural model offers a good prediction of the laminar flame speed while reducing the computational time. Malik et al. have worked to design and train a deep neural network for the prediction of laminar flame speeds of hydrogen and propane with air, which has been developed and shows good prediction accuracy [27]. In the same perspective, Sven E. et al. [28] worked on the application and comparison of multiple machine learning techniques for the calculation of laminar burning velocity for hydrogen-methane mixtures. The techniques used in this work are generalized linear regression model, support vector machine, random Forest, and artificial neural networks. The performance of the ANN model is comparable to the well-established detailed reaction mechanism (GRI 3.0), and the detailed reaction mechanism produced slightly more accurate results. To attempt a generalization of the laminar burning velocity prediction for several fuels, Zhongyu W. et al. [29] have developed machine learning models for the prediction of the laminar flame speeds of hydrocarbon and oxygenated fuels. These works show that a machine learning model based on the Gaussian process regression algorithm has good accuracy and is fast enough for integration into large-scale computational fluid dynamics for combustion studies.

In combustion engines, the combustion processes appear in a turbulent regime, so the turbulence effect increases combustion speed, and then the thermal power per unit volume increases. The kinetic energy due to fluid movement is degraded by the generation of eddies dissipated by friction. Simultaneously, this causes an increase in the diffusion effects [30]. The direct consequence is that the combustion rate is much higher than that which would have been obtained in the light regime. It is therefore important to produce a model that can provide accurate simulation results.

In this paper, several machine learning models are implemented and compared for turbulent flame velocity prediction in the combustion inside a hydrogen-natural gas spark ignition engine. An important particularity of this work is that the input variables of the model were chosen among the variables directly measurable experimentally based on the opinion of experts in combustion in internal combustion engines and not on the usual approaches to dimensionality reduction on a dataset. This approach eliminates calculations of intermediate variables that are usually numerous, time-consuming, complex, and error-prone. It, therefore, facilitates the use of the model obtained through its integration into systems or software operating in real-time.

## 2 Experimental setup and data preprocessing

### 2.1 Experimental setup

**Fig. 1:**
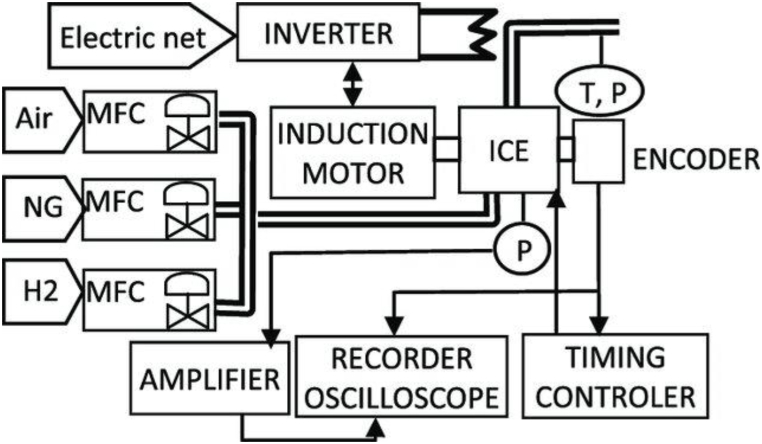
Experimental setup [30].

The data of this work was taken from a MINSEL 380, a 380-cc single-cylinder engine. It is a compression-ignition internal combustion engine that has been modified to become a spark ignition engine. The piston was machined axisymmetrically to reduce the compression ratio to 11.15. The experimentation and data acquisition were carried out by Blanca G. et al. [30], according to the device in Fig. 1. During the experiment, the ambient environment is under normal temperature and pressure conditions (Temperature = 298 K and Pressure = 01 Atm).

The different components of this experimental device are presented in Table 1.

**Table 1:** Instrumentation and equipment of the test bench.

Equipment	Manufacturer/Model	Objectives
**MFC (Mass Flow Controllers)**	- BROOKS/5851S	- Control Gas and H2 mass flow
	- BROOKS/5853S	- Control Air mass flow
**Induction Motor**	LEROY SOMER/PLS 180	Brake the Internal Combustion Engine
**Inverter**	FUJI FRENICMEGA 7.5 kW frequency inverter	Control Induction Motor
**ICE (Internal Combustion Engine)**	MINSEL 380, 380 cc single-cylinder	Produce Combustion Data
**Encoder**	AVL 360C/600 angular encoder with 0.6° resolution	Synchronizes the pressure signal
**Amplifier**	KISTLER 5018A	Condition the pressure signal
**Recorder Oscilloscope**	YOKOGAWA DL750	Record the Synchronized pressure signal
**P**	AVL GU21D sensor	Measure the chamber pressure
**T,P**	Exhaust Gas Temperature and Pressure sensor	Measure Exhaust Gas Temperature and Pressure
**Timing Controler**	–	Control the spark timing using the angular encoder signal

The experiments were carried out over more than 200 consecutive cycles and allowed the pressure data collection in the combustion chamber. Several fuels were used to have the dataset of this work.

- The natural gas: in this case the fuel-air equivalence ratio was varied in the range [0.7; 1] with a step of 0.1;
- The hydrogen: in this case the fuel-air equivalence ratio was varied in the range [0.7; 1] with a step of 0.1;
- The mixture of natural gas and hydrogen: for which the proportion of hydrogen varies from 0 % to 100 % with a step of 25 %. For each case, the fuel-air equivalence ratio value was calibrated to keep the temperature of the adiabatic flame constant. For 100 % natural gas and hydrogen tests, the lowest fuel-air equivalence ratio has been chosen, which guarantees stable engine behavior.

**Fig. 2:**
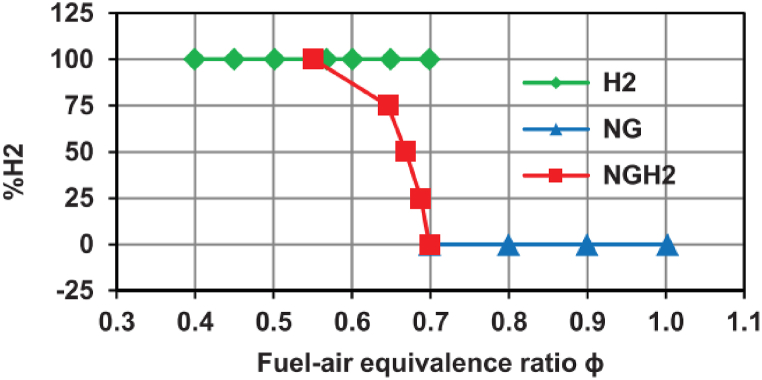
Test plan for each engine speed [30].

**Fig. 3:**
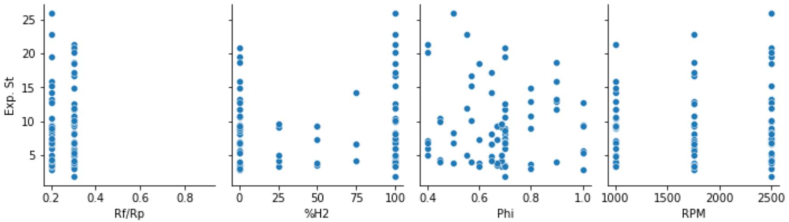
Ouput variable with each input variables

**Fig. 4:**
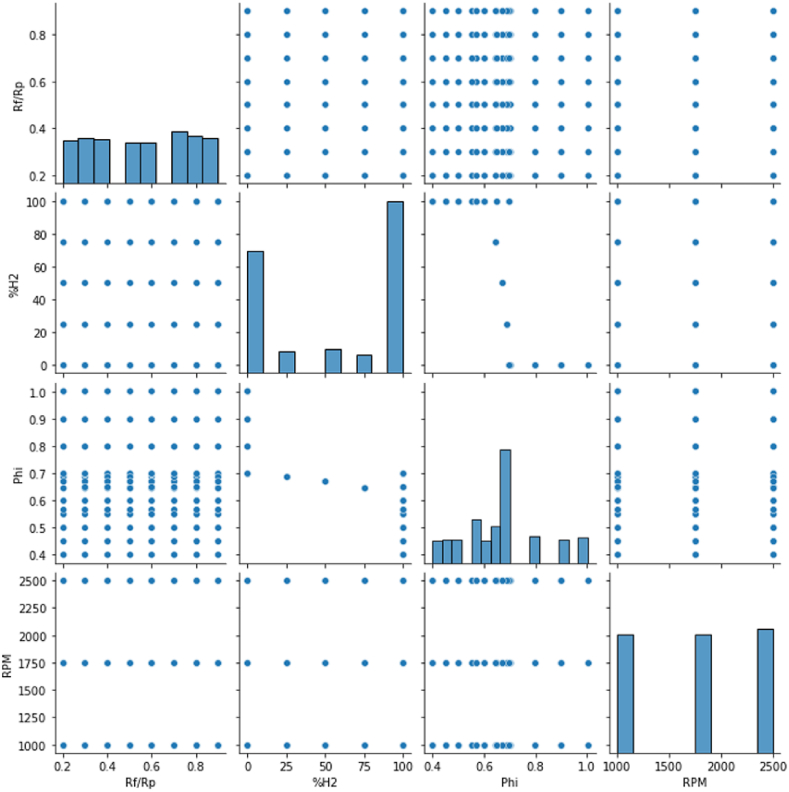
Visualization of input data

The tested points are shown in Fig. 2. For each test point, the experiment was reproduced for three engine speeds (1000 rpm, 1750 rpm, and 2500 rpm). Fig. 3, Fig. 4 respectively show relationship between input variables and relationship output variable and input variables.

### 2.2 Data preprocessing

Preprocessing is a very important step in the implementation of machine learning algorithms. It generally consists of purifying the data and also putting it in a form that guarantees the best efficiency of the machine learning tools chosen. The present work consisted mainly of a standardization operation. Standardization is a scaling method where the values are centered around the mean with a unit standard deviation. It guarantees the same scale for all the variables. This allows the algorithms to converge faster, improving their adaptability. The formula for standardization is:

$$Z_{i}=\frac{Z_{i}-{\bar{Z}}_{i}}{\sigma \left(Z_{i}\right)}$$

Where $Z_{i}$ corresponds to one of the features: Dimensionless flame front radius (R_f_/R_p_), Percentage of H2 (%H2), Fuel-air equivalence ratio (f), Engine Speed in rpm. Note that in our studies, only the feature followed the standardization step. The target variable (Experimental turbulent combustion Speed) remains the same. All these computations were performed using a Jupyter script with Scikit-Learn for the Linear regression and random forest models and TensorFlow for the neural network models. The architectures for each neural network will be describe when need arises.

### 2.3 Description of the models

In this section, the different machine learning techniques used are briefly presented.

**Multiple Linear Regression(MLR)** Multiple linear regression (MLR), sometimes called multiple regression for short, is a statistical technique that uses several explanatory variables to predict the outcome of a response variable. The parsimony principle motivates the use of the MLR algorithm. Indeed, MLR is the simplest algorithm to implement and it allows you to have an explicit model after training. The goal of multiple linear regression is to model the linear relationship between the explanatory (independent) variables and the response (dependent) variables. Multiple regression is a generalization of the least-squares interpolation technique because it involves more than one explanatory variable. They are formulated by the equation:

$$Y=a_{0}+a_{1}U_{1}+a_{2}U_{2}+\ldots ++a_{p}U_{p}+\varepsilon \text{.}$$

Here U_1_, U_2_, …, U_p_ stand for the independent variables or features and Y is the dependent variable or target.

**SupportVector Regression(SVR)** The Support Vector Machine (SVM) is a machine learning algorithm that implement the structural risk minimization inductive principle to obtain good generalizations on a limited number of learning patterns. The choice of the SVR algorithm is based on the fact that it is a versatile algorithm capable of modeling complex relationships through the use of different Kernel functions. In addition, it allows control over the trade-off between model error and complexity, despite being a black-box model. Support Vector Machines are directly derived from the framework provided by the Statistical Learning Theory, and they work by solving a constrained quadratic problem. The objective function for optimization is given by the combination of a loss function with a regularization term. Support Vector Regression is the most common application form of Support Vector Machine to solve regression problems. It is different from traditional linear regression methods as it finds a hyperplane that best fits the data points in a continuous space instead of fitting a line to the data points. This algorithm is interesting because it helps reduce the prediction error and allows support vector regression to handle non-linear relationships between input variables and the target variable using a kernel function. Support vector machines therefore prove to be very effective tools for regression problems where there may be complex relationships between the input variables and the target variable. The goal has been to find a function f(x) that has at most *ε* deviation from the obtained targets y_i_ for all the training data and is at the same time as flat as possible.

$$f\left(u\right)=\sum_{i=1}^{N}\left(\alpha_{i}-\alpha_{i}^{*}\right)k\left(u_{i},u\right)+b$$

where.

- $\alpha_{i},\alpha_{i}^{*}$ are Lagrange multipliers,
- The kernel function $k\left(u_{i},u\right)$ has been defined as a linear dot product of the nonlinear mapping: $k\left(u_{i},u\right)=\varphi \left(u_{i}\right)\varphi \left(u\right)$.
- φ is the mapping function.

**Fig. 5:**
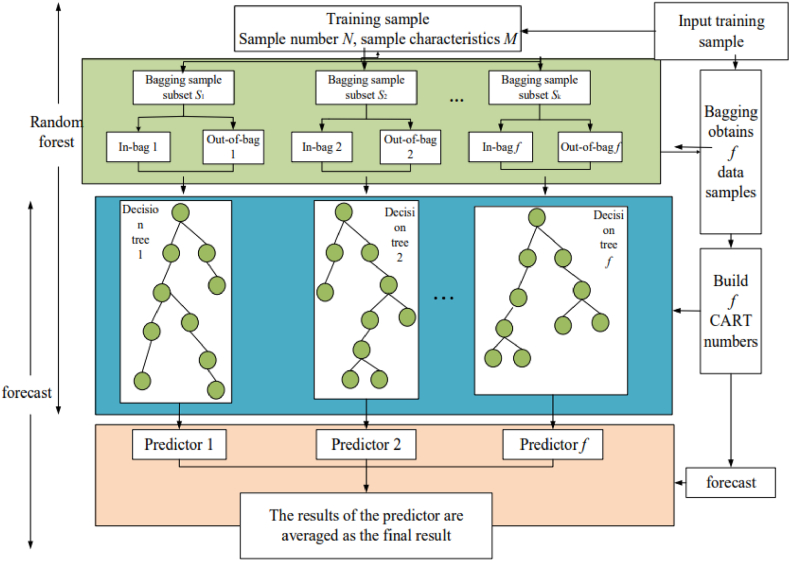
Random Forest training process

**Random Forest(RF)** The random forest algorithm is a classification algorithm that reduces the variance of predictions from a single decision tree, thus improving their performance. The Random Forest algorithm is known to provide good results with minimal parameter adjustments. Additionally, by averaging the results of multiple decision trees, it reduces the risk of model overfitting. To realize that, it uses a bagging type approach to combine many decision trees. It is used widely in Classification and Regression problems. Random forests have the advantage of giving good results for large datasets, they are very simple to implement and have few parameters. The Random Forest Algorithm can handle the data set containing continuous variables, as in the case of regression, and categorical variables, as in the case of classification. The Random Forest (RF) is a generalization of Bagging's idea on the basis of the decision tree algorithm and Ensemble learning algorithm [31]. Using the CART decision tree as the weak learner and building on the use of the decision tree, the following improvements are made to the decision tree: for a general decision tree, the best feature will be selected from the n sample features at a node as the left and right subtrees of the decision tree. In contrast, the Random Forest randomly selects some sample features from the nodes and computes the best feature to complete the split of the left and right subtrees of the decision tree. This can avoid certain overfitting features and eliminate any pruning of the decision tree, resulting in high accuracy and generalization performance of the overall model results. The RF structure is illustrated in Fig. 5.

The construction steps of the RF are as follows.

1. The Bagging sampling method is used to extract k data subsets (S_i_, i = 1, 2, …, k) from the original data set S, and in this k times of extraction, the data not extracted each time constitute k out of pocket data sets, and the extracted data sets are called in-pocket data sets.
2. Randomly select m* attributes from m features as a sub dataset, and then select the optimal feature from that subset for partitioning to construct a CART decision tree.
3. Each CART decision tree grows to its maximum degree without any pruning operation, and the value of m remains constant.
4. In total, k CART decision trees are generated for each of the k extractions, and each tree does not influence each other and exists independently.
5. The generated decision trees are integrated to form a Random Forest, and the average of the output values of all decision trees is taken as the final prediction value of the Random Forest.

**Fig. 6:**
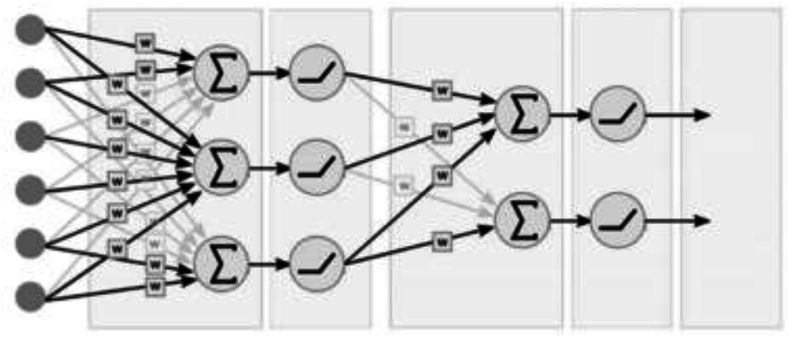
A feedforward neural network fully connected

**Artificial Neural Network (ANN)** The artificial neural network consists of layers of neurons, the first layer being the input layer, the last layer being the output layer, and the hidden layers between the input and output layers (See Fig. 6). The use of neural networks is mainly based on their ability to efficiently learn and model complex non-linear relationships between input and output variables. They also have good adaptability to changing data, a good capacity for generalization and a high tolerance for errors.

Each neuron can be considered as a calculation unit within which two main operations are carried out.

- An affine combination of input values with the weights of the upstream connections to which a bias is associated;
- An activation function into which the result of the affine combination is passed to obtain the output of the neuron.

Then, each successive layer n + 1 is related to the preceding layer n by:

$$U_{i}^{n+1}=f\left(\sum_{i=1}^{N}w_{i}^{n}U_{i}^{n}+b_{i}^{n}\right)$$

In the above equation*, U* is the vector in a given layer, *w* is the matrix containing the weights, vector *b* contains the biases, *N* is the number of neurons in the layer and *f* is the activation function. In the following cases an ANN was generated with a varied number of hidden layers and its own input and output layer.

The networks of the signal are transmitted using the activation function this depending on the hidden neuron's layers. An important challenge in the construction of artificial neural networks is the choice of hyperparameters (number of hidden layers, number of neurons per layer, activation functions, error calculation strategies, etc.). In this work, this choice was made through a trial-and-error approach.

The activation functions for the input and the hidden layers are the rectified linear unit (ReLU) and for the output layer is the linear function. Note that the rectified linear unit is defined by:

$$\text{ReLU}\left(u\right)=\left\{ \begin{array}{c} u\;if\;u\geq 0\\ 0\;if\;u<0 \end{array}\right.$$

These networks ANN were trained for 2000 to 5000 epochs. In the input layer, the features (Dimensionless flame front radius, Percentage of H2, Fuel-air equivalence ratio, Engine Speed in RPM, Intensity of isotropic turbulence (m/s), Laminar combustion speed (m/s), Thermal diffusivity of unburned, Cinematic viscosity of unburned, Prandtl number) are processed and in the output layer the target (Experimental turbulent combustion Speed) is calculated and compared to the training data set.

## 3 Results and discussion

This section presents the results obtained for the different models trained on the one hand with raw data and on the other hand with preprocessed data. For each model, we regression plot of the target variable obtained with the test data that have not been used for the training step.

### 3.1 Multiple linear regression (MLR) model

**Fig. 7:**
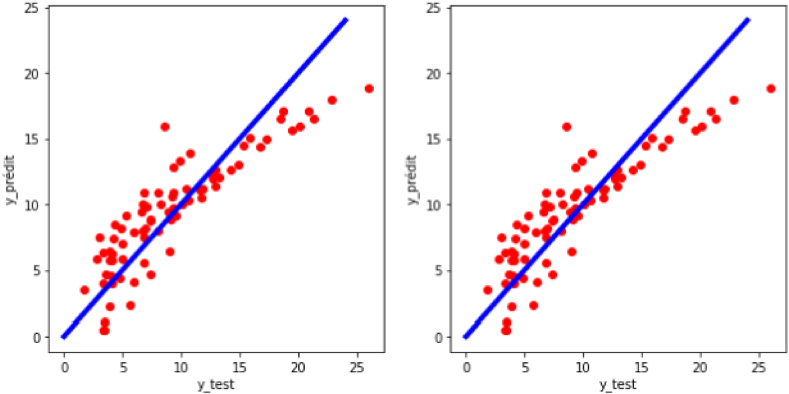
Regression plot of the target variable (y: experimental turbulent combustion speed) for the case of multiple linear regression.

The Multiple Linear Regression Model is the approach that produced the least accurate model in this work. Fig. 7 is the regression plot of the target variable (y: experimental turbulent combustion speed) for the case of multiple linear regression. One can see a great similarity in terms of precision and distribution of predicted values compared to actual values (R^2^ = 0.7749; RMSE = 2.5881) between the model with raw data (R^2^ = 0.7749; RMSE = 2.5881) and the model with preprocessed data. It reflects that the multiple linear regression algorithm could be poorly sensitive to differences in scale between the model variables.

### 3.2 Support vector regression (SVR) model

**Fig. 8:**
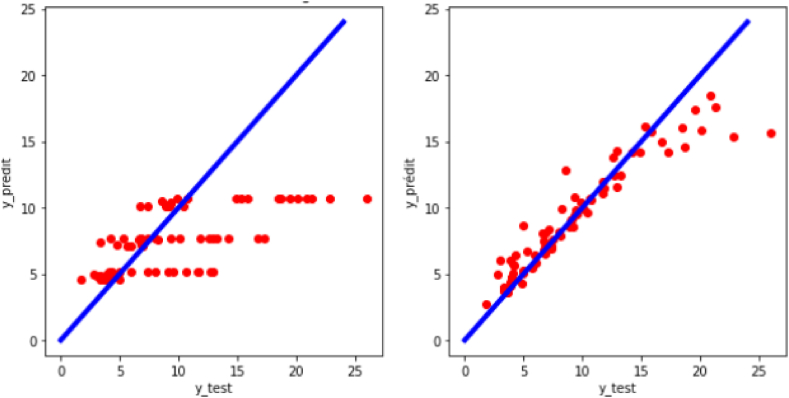
Regression plot of the target variable (y: experimental turbulent combustion speed) for the case of support vector regression.

Unlike the MLR model, the support vector regression model presents a dissimilarity between the results from training with raw data (R^2^ = 0.2615; RMSE = 4.6888) and the results with preprocessed data (R^2^ = 0.8558; RMSE = 2.0715). Indeed, scaling (standardization) of the data seems necessary to increase the model's effectiveness. Thus, with standardized data, we obtain a better result compared to the MLR model (See Fig. 8).

Due to its simplicity of implementation, this model can be used in applications that do not require very high precision.

### 3.3 Random forest (RF) model

The random forest model is the one that presented the best performance in this work.

**Fig. 9:**
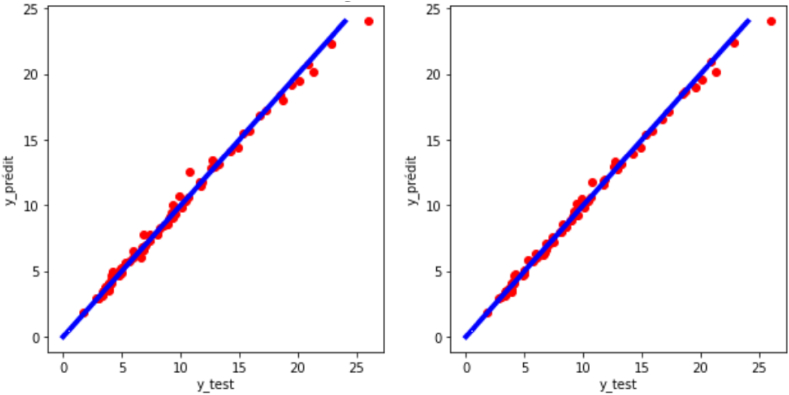
Regression plot of the target variable (y: experimental turbulent combustion speed) for the case of random forest.

Fig. 9shows that the random forest model correctly fits the dataset and allows a reliable and efficient prediction of experimental turbulent combustion speed both for raw data (R^2^ = 0.9932; RMSE = 0.4507) and for preprocessed data (R^2^ = 0.9939; RMSE = 0.4274).

This model can therefore be used for the calculation of experimental turbulent combustion speed in high-precision applications without requiring transformations on the variables.

### 3.4 Artificial neural network (ANN) model

Several neural network architectures have been tested with different numbers of hidden layers, numbers of neurons per layer, activation functions, error calculation strategies, etc.

**Fig. 10:**
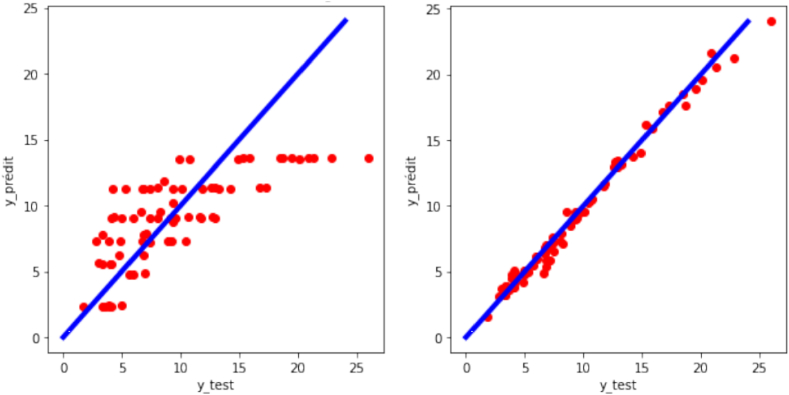
Regression plot of the target variable (y: experimental turbulent combustion speed) for the case of artificial neural network.

The architecture implemented in this paper is a multi-layer feed-forward neural network (MFNN), which for the target consists of three layers (input layers with 7 neurons, hidden layers with 128 neurons, and output layers with 1 neuron), and for the other consists of four layers (input target layers with 7 neurons, two hidden layers with 256 neurons for each, and an output layer with one neuron) because it gives very precise results with reasonable complexity criteria. With an R^2^ = 0.9882 and an RMSE = 0.5918, it is the second most precise model (on preprocessed data) presented in this work. However, we note that on raw data, its effectiveness is greatly reduced; this model is highly sensitive to differences in scale in the data (See Fig. 10).

### 3.5 Synthesis and discussion

Table 2, Table 3 give a summary of the results (evaluation metrics and cost function for each method implemented) presented in the previous sections, respectively, for original data and preprocessed (standardized) data.

**Table 2:** Summary of results obtained original data.

Method	R^2^ Value	RMSE Value
**Multiple Linear Regression**	0.7749	2.5881
**Support Vector Regression**	0.2615	4.6888
**Random Forest**	0.9932	0.4507
**Artificial Neural Network**	0.6058	3.4253

**Table 3:** Summary of results obtained standardized data.

Method	R^2^ Value	RMSE Value
**Multiple Linear Regression**	0.7749	2.5881
**Support Vector Regression**	0.8558	2.0715
**Random Forest**	0.9939	0.4274
**Artificial Neural Network**	0.9882	0.5918

It could be concluded, like in the work of Sven E. et al. [28], that the ANN and Random Forest models give the most accurate results. However, the Random Forest Model gives the best results and remains easier to implement, unlike the neural network, which generally requires several attempts to get hyperparameters from the optimized ANN. With a forecasting accuracy of over 90 %, both approaches can make reasonable predictions for most industrial applications. The varying of the performance levels of the models can be explained by their ability to found complexes nonlinear relationship between the inputs variables and output variable.

The comparison of the calculation times (t_c_) of the different machine learning models with forecasts of a detailed reaction mechanism (DRM) was then investigated. The GRI 3.0 [32] has been selected as the representative mechanism for hydrogen methane flames (See Table 4).

**Table 4:** Comparison of calculation time.

Method	Calculation time t_c_(s)
**GRI 3.0 detailed mechanism + Blanca G. et al correlation model for turbulent speed** [12]	_∼_39 782
**Multiple Linear Regression**	<1
**Support Vector Regression**	<1
**Random Forest**	<1
**Artificial Neural Network**	_∼_6.42

All the models obtained have a significantly lower calculation time than the reaction mechanism GRI3.0. This can be explained by the fact that the reaction mechanism GRI3.0 integrates several complex sub-models based on the resolution of transport and thermal equations (partial differential equations), which are generally computationally intensive. However, these models remain less robust than the GRI3.0 reaction mechanism, i.e., it remains quite difficult to build a machine learning model that effectively calculates the turbulent flame speed for several types of fuels.

## 4 Conclusion

This paper focused on the application and comparison of multiple machine-learning techniques for computing the turbulent combustion velocity for hydrogen-natural gas spark ignition engines. The results show that machine learning algorithms allow a significant time saving (around 11 h for the calculation carried out in this work) compared to usual approaches using thermal and transport equations. The results show that preprocessed data give the best result. The evaluation metrics give respectively R^2^ = 0.9882 and RMSE = 0.5918 for ANN, R^2^ = 0.9939 and RMSE = 0.4274 for Random Forest, R^2^ = 0.7749 and RMSE = 2.5881for MLR and R^2^ = 0.8558 and RMSE = 2.0715 for SVR. The ANN and the Random Forest models gave the most accurate results. However, the Random Forest model gives the best results (both for original data and for preprocessed data) and remains easier to implement. The results of this work can therefore be integrated into CFD software as an alternative to turbulent combustion velocity calculation sub-models or can be used for designing engine monitoring and control systems, firefighting systems, post-combustion systems, simulation, and prototyping combustion engines, etc. In addition, the choice of using directly measurable model input data facilitates the deployment of these models to systems operating in real-time. One approach to improving this work would be to produce machine learning models for computing turbulent combustion velocity for different fuel types.

## CRediT authorship contribution statement

**Nelson Junior Issondj Banta:** Writing – review & editing, Writing – original draft, Visualization, Methodology, Formal analysis, Data curation, Conceptualization. **Njionou Patrick:** Writing – review & editing, Software, Investigation, Formal analysis, Data curation. **Florence Offole:** Validation, Supervision, Resources, Project administration, Methodology, Conceptualization. **Ruben Mouangue:** Validation, Supervision, Project administration, Methodology, Conceptualization.

## Declaration of competing interest

The authors declare that they have no known competing financial interests or personal relationships that could have appeared to influence the work reported in this paper.
